# Challenge of Innovative Technology: How to Improve Efficiency of Korean Medicine?

**DOI:** 10.1155/2016/8201852

**Published:** 2016-11-20

**Authors:** Wansu Park, Salih Mollahaliloglu, Vitaly Linnik, Han Chae

**Affiliations:** ^1^Department of Pathology, College of Korean Medicine, Gachon University, Seongnam, Republic of Korea; ^2^Department of Public Health, Faculty of Medicine, Yildirim Beyazit University, Ankara, Turkey; ^3^Social Insurance Fund of Russian Federation, Moskva, Russia; ^4^Division of Longevity and Biofunctional Medicine, School of Korean Medicine, Pusan National University, Busan, Republic of Korea

Advancement in science inevitably brings about the development of medicine. With the state-of-the-art technology, Western medicine continuously innovates. Nevertheless, there are still global health issues to be resolved, that is, chronic pain disease, chronic inflammatory disease, autoimmune disease, chronic sprain, myofascial pain syndrome, ischemic stroke, dementia, Parkinson's disease, arteriosclerosis, obesity, metabolic disease, type 2 diabetes, vertigo, allergy, infertility, dysmenorrhea, endometriosis, degenerative arthritis, and cancer. Additionally, new emerging infectious diseases such as* E. bola*, MERS, and bird flu as well as antibiotic resistant infections have become a threat to humans. Thus, not less than other traditional and integrative medicines, Korean Medicine (KM) is also challenged to innovatively cope with modern health issues. In 2014, the National Health Insurance of Korea covered 55% of total cost which patients (over 13 millions) paid for medical services provided by KM facilities including hospitals and clinics. More detailed, accurate, and valuable data regarding KM (including clinical and basic study) are needed not only to deal efficiently with modern health issues but also to bring about the innovation of KM services. To improve efficiency and accuracy in KM, modern technology and bioscience are indispensable. The innovation of practice and evolvement in KM help people overcome diseases and live healthily.

Our special issue, which had opened for 6 months in the second half of 2015, focused on challenge of innovative technology: how to improve efficiency of KM.

An article by D.-H. Kim et al. described that they took the TEM images of a cross section of the primo vessel (PV) inside a lymph vessel; the TEM study reveals the loosely distributed collagen fibers with plenty of empty spaces and the lumens with the endothelial nuclei; it turns out to be very similar to the ultrastructure of the PVs observed on the surfaces of internal organs; it also shows how compactly the PV is surrounded with lymphocytes.

J.-H. Kim et al. reported that the identification of* Atractylodes* rhizomes samples, authenticated by their morphological features as* Atractylodes japonica* Koidz. (Changchul and Baekchul),* Atractylodes chinensis* Koidz., and* Atractylodes macrocephala* Koidz., is confirmed as* Atractylodes japonica*,* Atractylodes chinensis*, and* Atractylodes macrocephala* by internal transcribed spacer sequencing; the results from chemometric analyses show that the chemical components of the crude drugs from* Atractylodes japonica* are significantly different from those from* Atractylodes macrocephala* but are similar to those from* Atractylodes chinensis* according to the results from chemometric analyses; the categorization by age of* Atractylodes japonica* as Changchul or Baekchul is not recommended. The results indicate that* Atractylodes japonica* should be categorized as “Changchul” and should not be further categorized by age.

A study by H.-Y. Cha et al. described that they investigated the preventive therapeutic effects of Hataedock (HTD) treatment on inflammatory regulation and skin protection in AD-induced NC/Nga mice under high-fat diet conditions; HTD downregulates the levels of IL-4 and PKC but increases the levels of LXR; HTD also suppresses the mast cell degranulation and release of MMP-9, substance P; the levels of TNF-*α*, p-I*κ*B, iNOS, and COX-2 are also decreased; the upregulation of inflammatory cell's apoptosis is confirmed as increase of apoptotic body and cleaved caspase-3 and decrease of Bcl-2; HTD also reduces edema, angiogenesis, and skin lesion inflammation; HTD suppresses various inflammatory response on AD-induced mice with obesity through the regulation of Th2 differentiation and the protection of lipid barrier.

W.-S. Jung et al. reported that Chunghyul-dan (CHD) is a herbal complex containing 80% ethanol extract and is composed of Scutellariae Radix, Coptidis Rhizoma, Phellodendri Cortex, Gardeniae Fructus, and Rhei Rhizoma; CHD has shown antilipidemic, antihypertensive, antiatherosclerotic, and inhibitory effects on ischemic stroke recurrence with clinical safety; the antilipidemic effect of CHD results from 3-hydroxy-3-methylglutaryl-coenzyme A reductase and pancreatic lipase-inhibitory activity; the antihypertensive effect likely results from the inhibitory effect on endogenous catecholamine(s) release and harmonization of all components showing the antihypertensive effects; anti-inflammatory and antioxidant effects on endothelial cells are implicated to dictate the antiatherosclerotic effects of CHD; CHD also shows neuroprotective effects on cerebrovascular and parkinsonian models; CHD could be helpful for the prevention of the recurrence of ischemic stroke; CHD could be a promising medication for treating and preventing cerebrovascular and cardiovascular diseases.

An interesting study by Y.-K. Song et al. evaluated that the serum C-peptide, insulin, leptin, lipocalin-2, and adipsin levels in the obese female Korean adults group are significantly higher than in the normal female Korean adults group; mean serum leptin of Eui-E-In-Tang group, which was randomized to receive Eui-E-In-Tang for 12 weeks, is significantly reduced at the end point of 12 weeks; Eui-E-In-Tang is composed of Coicis Semen, Angelicae Gigantis Radix, Atractylodis Rhizoma Alba, Ephedrae Herba, Cinnamomi Ramulus, Paeoniae Radix, and Glycyrrhizae Radix; Eui-E-In-Tang may exert immunomodulatory effect via reducing the circulating concentration of leptin in obese female Korean adults.

C. Yang et al. reported that they collected 166 injury cases from 94 Korean male and female national volleyball players; knee (25.9%), low back (13.3%), elbow, and ankle (8.4%) injuries are most common; joint (41.6%) and muscle (30.7%) are major injured tissues; KM team medical doctors utilize acupuncture (40.4%), chuna manual therapy (16.0%), physical therapy (15.2%), taping (9.0%), and cupping (7.8%) to treat volleyball injuries; any type of medications is used infrequently; additional physical and exercise therapies are preferred after receiving acupuncture (both 46.9%); injury and treatment parameters in this study could be useful to build advanced KM model in sport medicine.

In conclusion, we expect that this special issue updates innovative technologies in KM and makes useful progress for improving efficiency and accuracy in KM.

